# Tea Chrysanthemum Detection by Leveraging Generative Adversarial Networks and Edge Computing

**DOI:** 10.3389/fpls.2022.850606

**Published:** 2022-04-07

**Authors:** Chao Qi, Junfeng Gao, Kunjie Chen, Lei Shu, Simon Pearson

**Affiliations:** ^1^College of Engineering, Nanjing Agricultural University, Nanjing, China; ^2^Lincoln Agri-Robotics Centre, Lincoln Institute for Agri-Food Technology, University of Lincoln, Lincoln, United Kingdom

**Keywords:** tea chrysanthemum, generative adversarial network, deep learning, edge computing, NVIDIA Jetson TX2

## Abstract

A high resolution dataset is one of the prerequisites for tea chrysanthemum detection with deep learning algorithms. This is crucial for further developing a selective chrysanthemum harvesting robot. However, generating high resolution datasets of the tea chrysanthemum with complex unstructured environments is a challenge. In this context, we propose a novel tea chrysanthemum – generative adversarial network (TC-GAN) that attempts to deal with this challenge. First, we designed a non-linear mapping network for untangling the features of the underlying code. Then, a customized regularization method was used to provide fine-grained control over the image details. Finally, a gradient diversion design with multi-scale feature extraction capability was adopted to optimize the training process. The proposed TC-GAN was compared with 12 state-of-the-art generative adversarial networks, showing that an optimal average precision (AP) of 90.09% was achieved with the generated images (512 × 512) on the developed TC-YOLO object detection model under the NVIDIA Tesla P100 GPU environment. Moreover, the detection model was deployed into the embedded NVIDIA Jetson TX2 platform with 0.1 s inference time, and this edge computing device could be further developed into a perception system for selective chrysanthemum picking robots in the future.

## Introduction

Some researches indicated that tea chrysanthemum has great commercial value (Liu et al., 2019). Besides, tea chrysanthemums offers a range of health benefits (Yue et al., 2018). For instance, it can considerably suppress carcinogenic activity and has significant anti-aging effects (Zheng et al., 2021). In the field, a tea chrysanthemum plant could present multiple flower heads, varying in different growth stages and sizes. Normally, tea chrysanthemums at the early flowering stage hold the best commercial value and health benefits, so they are mainly manually harvested at the early flowering stage, and this is a labor-intensive and time-consuming process.

Rapid developments in artificial intelligence and robotics offer a new opportunity to automate this harvesting task, dealing with the current scarcity of the skilled laborers (Dhaka et al., 2021; Kundu et al., 2021; Liu et al., 2021; Wieczorek et al., 2021). Hence, it is urgent to develop a selective harvesting robot. The perception system and manipulator are the two key components for developing selective harvesting robot. Many studies have shown that a high resolution image dataset has a profound impact on detection performance as it contains fine-grained features for object recognition (Zhou et al., 2021). However, collecting a dataset of tea chrysanthemums presents inherent difficulties. Tea chrysanthemums normally mature once a year and have to be picked at the early flowering stage to maximize commercial values. Moreover, the early flowering stage is incredibly short, typically from only 2 days to 1 week. Currently, there is no publicly available dataset on tea chrysanthemums worldwide for developing a detection algorithm, which is a hindrance to build an intelligent selective harvesting robot and other intelligent phytoprotection equipment (Ansari et al., 2020; Alsamhi et al., 2021, 2022), e.g., Internet of Things based solar insecticidal lamps. Therefore, it is important to have a good dataset of tea chrysanthemums.

Using classical data augmentation to expand datasets and balance categories were reported in Tran et al. (2021). Nevertheless, classical data enhancement methods (rotation, translation, flipping, and scaling, etc.) only allow for restricted feature diversity, prompting the utilization of generated data. Generated samples provide more variation and further enrich the dataset to improve training accuracy. Recent approaches address the data generation issues through utilizing generative adversarial networks (GANs) (Wang et al., 2019). These methods use an encoder-decoder strategy to generate fake images that can be used to enrich the original dataset. GANs have shown the impressive results by generating stunning fake images such as human faces (Zhao et al., 2019). However, GANs still suffer from non-negligible flaws. In our case, three issues need to be further investigated.

Issue 1: In the current agricultural field, GAN generates images with a maximum resolution of 256 × 256 pixels. This is not suitable for the chrysanthemum detection task as the low resolution images contain restricted information about the environment related features, which somewhat affects the robustness of the whole model. How to generate images that can meet the detection task resolution of the tea chrysanthemum is an issue requiring further exploration.

Issue 2: The traditional GAN directly provides the latent code to the generative network, resulting in a massive feature entanglement, thus directly influencing the diversity of the generated chrysanthemum images. How to design a network structure that could improve the diversity of the generated chrysanthemum images is an issue to be further explored.

Issue 3: The alternating optimization of generators and discriminators makes the GAN prone to pattern collapse and gradient vanishing during training, so how to achieve stable training is an issue to be further explored.

Based on these three issues, we propose a tea chrysanthemum – generative adversarial network (TC-GAN) that can generate images with diversity at 512 × 512 resolution, as well as stable training. We decouple the latent code into intermediate vectors via a Mapping Network, resulting in controlling the diversity of chrysanthemum features. Also, we apply path length regularization in the Mapping Network, leading to more reliable and consistent behavior of the model and making architectural exploration easier. In the generative network, we add Stochastic variation after each convolutional layer to increase the diversity of the chrysanthemum images. Finally, we embed Res2Net into the generative network so that we can better guide the gradient flow to alleviate pattern collapse and gradient vanishing during the training process.

**FIGURE 1:**
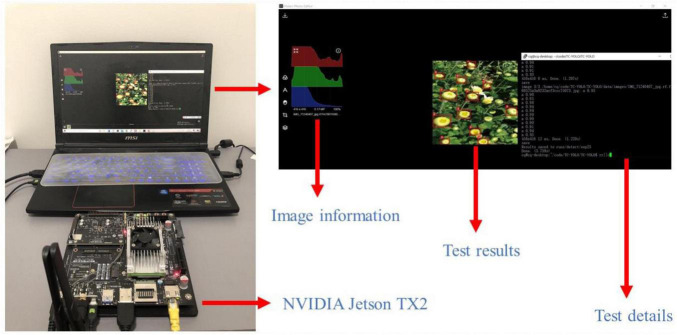
The results of testing tea chrysanthemum – generative adversarial network (TC-GAN) on NVIDIA Jetson TX2. First, we used an HDMI cable to connect the laptop with the Jetson TX2, and ensure that the laptop and Jetson TX2 were under the same wireless network. Then, the TC-YOLO model and the tea chrysanthemum dataset were embedded in the flashed Jetson TX2 for testing.

In this article, our goal is to generate datasets that can be used for the tea chrysanthemum detection task. We tested the images generated by TC-GAN on some state-of-the-art object detection models, as well as our own proposed detection model (TC-YOLO) (Qi et al., 2022). Moreover, for subsequent development work on an automated selective chrysanthemum picking robot, we chose to test the images generated by TC-GAN on a low-power embedded GPU platform, the NVIDIA Jetson TX2, as shown in Figure 1.

The contributions of this article are as follows:

1. High resolution (512 × 512) images of tea chrysanthemums with complex unstructured environments (illumination variations, occlusions, overlaps) were generated using the proposed TC-GAN model.
2. The images generated with TC-YOLO quantified the impact of five aspects, i.e., (1) dataset size, (2) epoch number, (3) different data enhancement methods, (4) various object detection models, and (5) complex unstructured environments on the TC-YOLO model, and verified the superiority of the TC-GAN model by comparing with some state-of-the-art GANs.
3. TC-YOLO, developed from images generated by TC-GAN, was successfully deployed and tested in the edge device NVIDIA Jetson TX2.

The rest of this article is organized as follows. Section “Related Work” describes the research background. Section “Materials and Methods” depicts the proposed TC-GAN structure. Section “Results” presents the experimental details. Section “Discussion” describes the contribution of this article and the limitations of the research, as well as pointing out possible future solutions. Section “Conclusion” gives a concise summary of this article.

## Related Work

Some GANs emerged to response the aforementioned Issue 3. Conditional Generative Adversarial Net (CGAN) (Liu et al., 2020) can strengthen the robustness of the model by applying conditional variables to the generator and discriminator that alleviate pattern collapse. Deep convolutional generative adversarial networks (DCGAN) (Jeon and Lee, 2021), the first GAN architecture based on convolutional neural networks, demonstrates a stable training process that effectively mitigates pattern collapse and gradient vanishing, but suffers from low quality and inadequate diversity of the generated images. Wasserstein GAN (WGAN) (Zhou et al., 2022) uses Wasserstein as an alternative to Jensen-Shannon (JS) divergence for comparing distributions, producing better gradients and improving training stability. Nevertheless, WGAN has difficulty converging due to the use of weight clipping, which can lead to sub-optimal performance.

Conditional Generative Adversarial Net, DCGAN, and WGAN had a profound impact on the development of GAN. Moreover, with the development of deep learning techniques, some high-performance GANs emerged to mitigate pattern collapse and gradient vanishing, resulting in stable training. Specific details are shown in Table 1. We will compare these models with the proposed TC-GAN in section “Results.”

**TABLE 1:** Details of the twelve latest generative adversarial networks.

Algorithm	Published year	Characteristic	Resolution
Progressive GAN (Collier et al., 2018)	2017	Grow the generator and discriminator progressively	64 × 64
LSGAN (Mao et al., 2019)	2017	Applying the least squares loss function	112 × 112
SN-GAN (Mufti et al., 2019)	2018	Applying spectral normalization	32 × 32
MGAN (He et al., 2019)	2018	Applying multi-channel gait templates	64 × 64
Dist-GAN (Tran et al., 2018)	2018	Applying a latent-data distance constraint	64 × 64
Rob-GAN (Liu and Hsieh, 2019)	2019	Jointly optimize generator and discriminator	128 × 128
AutoGAN (Gong et al., 2019)	2019	Applying NAS algorithm	64 × 64
BigGAN (Qiao et al., 2020)	2018	Applying orthogonal regularization	512 × 512
Improved WGAN (Yang et al., 2020)	2020	Injecting an instance noise	128 × 128
Improved WGAN-GP (Kim et al., 2021)	2021	Wasserstein GAN with gradient penalty	28 × 28
Improved DCGAN (Chao et al., 2021)	2021	Applying batch normalization	64 × 64
DAG (Tran et al., 2021)	2021	Improve learning of the original distribution	48 × 48

We collated the available literature on image recognition using GANs in agriculture, with a particular focus on the generated image resolution and the complex unstructured environment in the generated images, as shown in Table 2. High-resolution images contain better fine-grained features and more complex unstructured environments, facilitating the extraction of abundant image features for robust detection results. Also, high resolution images make transfer learning easier, and current object detection frameworks typically require datasets with a resolution higher than 416 × 416 (Liu and Wang, 2020). Not only that, to summarize the GANs in Tables 1, 2, several structural improvements are needed. First, the latent codes (input vectors) in the GANs in Tables 1, 2 are directly fed into the generator network. Nevertheless, the design of using latent codes to generate specific visual features is somewhat restricted so that it has to consider the probability density of the input data. This design can prevent some latent codes from being mapped to features, resulting in feature entanglement. The proposed model structure allows vectors to be generated without considering the input data distribution through a custom mapping network, as well as reducing the correlation between different features. Second, multi-scale extraction and feature fusion can effectively guide the gradient flow, but in the GANs in Tables 1, 2, the structure is designed mainly for normalization approaches, loss functions, control variables and mapping relationships between generators and discriminators. Currently, the structure of GANs lacks design for multi-scale extraction and feature fusion. The generator structure of the proposed model focuses on the combination of multi-scale extraction and feature fusion.

**TABLE 2:** Available literature using GAN for image recognition in agriculture.

Algorithm	Published year	Task	Accuracy (%)	Resolution	Test environment
DCGAN (Gandhi et al., 2018)	2018	Plant disease detection	88.6	64 × 64	Illumination
C-DCGAN (Hu et al., 2019)	2019	Tea leaf’s disease identification	90	64 × 64	Illumination
DCGAN (Douarre et al., 2019)	2019	Apple scab segmentation	60	28 × 28	Ideal
CycleGAN (Padilla-Medina et al., 2019)	2019	Detection of apple lesions in orchards	95.57	64 × 64	Ideal
DCGAN (Bian et al., 2019)	2019	Tea clones identifications	76	64 × 64	Ideal
Deep CORAL (Marino et al., 2020)	2020	Potato defects classification	90	64 × 64	Ideal
CAAE (Zhong et al., 2020)	2020	Citrus plant diseases recognition	53.4	64 × 64	Illumination
DCGAN (Nafi and Hsu, 2020)	2020	Plant disease detection	86.63	64 × 64	Ideal
BEGAN (Luo et al., 2020)	2020	Pine cone detection	95.3	64 × 64	Ideal
CGAN (Olatunji et al., 2020)	2020	Kiwi geometry reconstruction	75	28 × 28	Ideal
DCGAN (Talukdar, 2020)	2020	Plant disease classification	95.88	64 × 64	Ideal
DCGAN (Hu et al., 2020)	2020	Recognition of diseased pinus trees	78.6	64 × 64	Ideal
TasselGAN (Shete et al., 2020)	2020	Plant traits detection	94	128 × 128	Illumination
CycleGAN (Zhao et al., 2021a)	2021	Bale detection	93	64 × 64	Ideal
DCGAN (Espejo-Garcia et al., 2021)	2021	Weeds identification	93.23	64 × 64	Ideal
DoubleGAN (Zhao et al., 2021b)	2021	Plant disease detection	99.06	64 × 64	Ideal
AR-GAN (Nazki et al., 2020)	2020	Plant disease recognition	86.1	256 × 256	Illumination

## Materials and Methods

### Datasets

**FIGURE 2:**
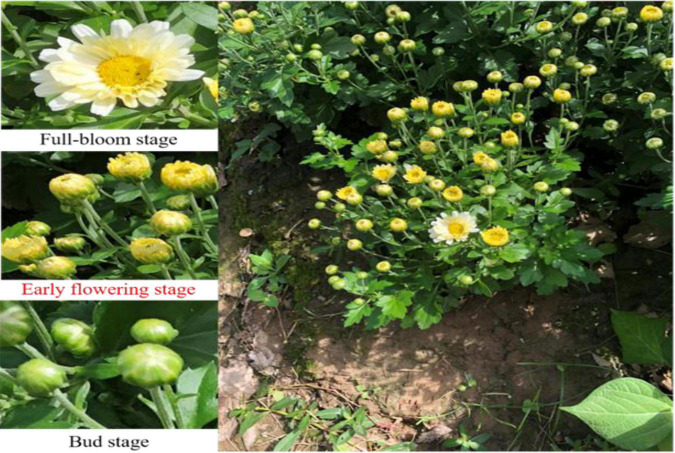
Examples of the collected original images.

The tea chrysanthemum dataset utilized in this article was collected from October 2019 to October 2020 in Sheyang County, Dongzhi County and Nanjing Agricultural University, China. The datasets were all collected using an Apple X phone with an image resolution of 1080 × 1920. The datasets were captured in natural light under three environments, including illumination variation, overlap and occlusion. The chrysanthemums in the dataset comprise three flowering stages: the bud stage, the early flowering stage and the full bloom stage. The bud stage refers to when the petals are not yet open. The early flowering stage means when the petals are not fully open and the full bloom stage denotes when the petals are fully open. The three examples of the original images are shown in Figure 2.

### NVIDIA Jetson TX2

**FIGURE 3:**
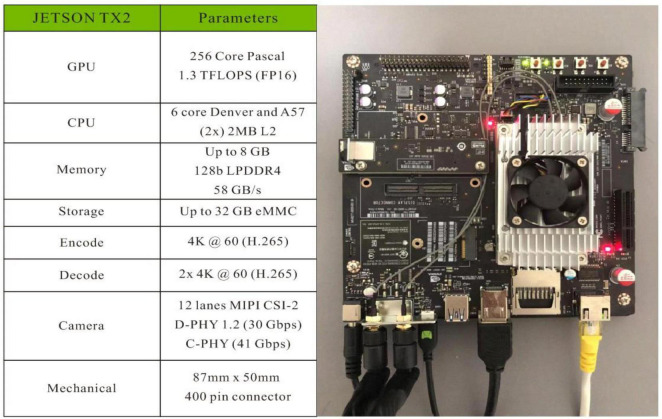
NVIDIA Jetson TX2 parameters.

There is no need to transmit all gathered image data back to cloud for further processing since the communication environment in countryside is generally not stable and the long time delay for smart equipment, i.e., chrysanthemum picking robot, is not acceptable. The NVIDIA Jetson TX2 has a 6-core ARMv8 64-bit CPU complex and a 256-core NVIDIA Pascal architecture GPU. The CPU complex consists of a dual-core Denver2 processor and a quad-core ARM Cortex-A57, as well as 8 GB LPDDR4 memory and a 128-bit interface, making it ideal for low power and high computational performance applications. Thus, this edge computing device was chosen to design and implement a real-time object detection system. We introduced the NVIDIA Jetson TX2 in Figure 3.

### Architecture

**FIGURE 4:**
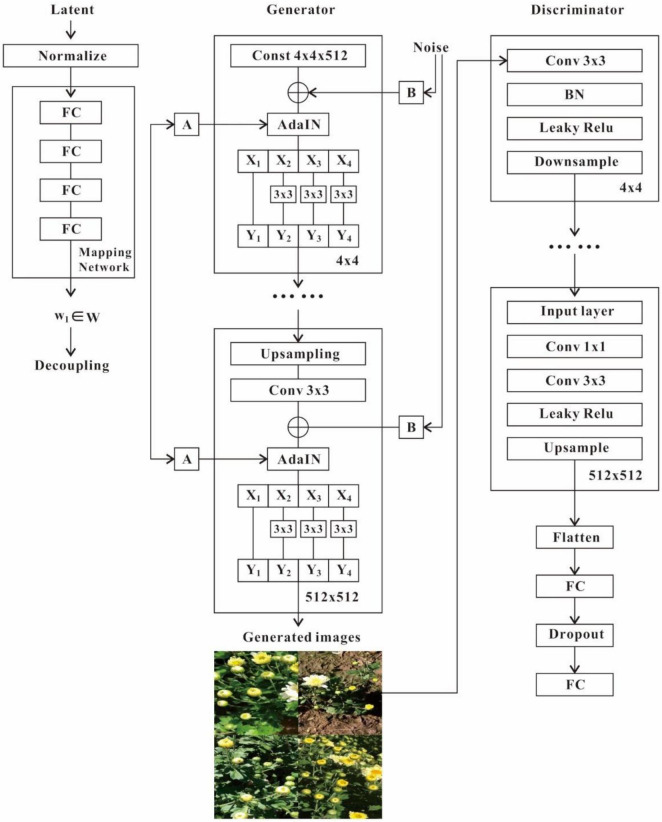
Structure of the proposed TC-GAN network. Mapping network can effectively capture the location of potential codes with rich features, benefiting the generator network to accurately extract complex unstructured features. A represents the learned affine transformation. B denotes the learned per-channel scaling factor applied to the noisy input. Discriminator network is designed to guide the training of the generator network, which is continuously confronted by alternating training between the two networks, ultimately enabling the generator network to better execute the generation task.

The proposed TC-GAN comprises a generator and a discriminator. In the generator, the non-linear mapping network *f* is implemented with a 4-layer multilayer perceptron (MLP), as well as applying path length regularization to decorrelate neighboring features for more fine-grained control of the generated images. The learned affine transform then specializes w to the style *y* = (ys, yb), controlling the Adaptive Instance Normalization (AdaIN) operation after each convolutional layer of the synthetic network *g*, followed by Res2Net to better guide the gradient flow without increasing the network computational workload. Finally, we introduce noisy inputs that enable the generator to provide random detail. We inject a specialized noise image into each layer (4^2^–512^2^) of the generator network, these are single channel images composed of Gaussian noise. The noise images are used with a feature scaling factor broadcast to all feature maps, and subsequently applied to the output of the corresponding convolution. Leaky ReLU is employed as the activation function throughout the generator. In the discriminator, the generated 512 × 512 resolution image and the real image of the same resolution are fed into the discriminator network simultaneously and mapped to 4 × 4 via convolution. In the whole convolution process, some diverse modules are inserted, including CL (Convolution + Leaky ReLU) and CBL (Convolution + Batch Normalization + Leaky ReLU). It is worth noting that the GAN training tends to be unstable, and no extra modules are inserted to guide the gradient flow and make the overall discriminator network look as simple as possible. Also, due to the lack of gradient flow in the underlying layer, the BN module was not inserted in the convolution process. Leaky ReLU is utilized as the activation function throughout the discriminator. Moreover, the generator and discriminator both employ the Wasserstein distance with gradient penalty as the loss function. The structure of TC-GAN is shown in Figure 4.

### Mapping Network

The mapping network consists of four fully connected layers that map the latent space *z* to the intermediate latent space w via affine transformations. Figure 4 depicts the structure of the mapping network. To capture the location of latent codes with rich features, this network encourages feature-based localization. A mixed regularization strategy is adopted, where two random latent codes are used instead of one latent code to generate some images during the training process. When generating an image, we simply switch from one latent code to another at a randomly picked point in the generative network. Specifically, the two latent codes *z*1, *z*2 are under control in the mapping network, and the corresponding *w*1, *w*2 are allowed to fix the features so that *w*1 works before the intersection point and *w*2 works after the intersection point. This regularization strategy prevents neighboring features from being correlated. Furthermore, extracting potential vectors in a truncated or reduced sample space helps to improve the quality of the generated images, although a certain degree of diversity in the generated images would be lost. Based on this, we can consider a similar approach. First, after training, intermediate vectors are generated in the mapping network by randomly selecting different inputs and calculating the center of mass in these vectors:


(1) $$\bar{w}=E_{z∼P\,\left(z\right)}$$

where $\bar{w}$ stands for the center of mass and *z* denotes the latent space.

We can then scale the deviation of a given *w* from the center as:


(2) $$w^{\prime }=\bar{w}+\psi \,\left(w-\bar{w}\right)$$

where *w*′ refers to the truncated *w* and ψ defines the difference coefficient between the intermediate vector and the center of mass.

### Stochastic Variation

The sole input of traditional networks is through the input layer, which generates spatially varying pseudo-random numbers from earlier activations. This method consumes the capacity of the network and thus makes it difficult to hide the periodicity of the generated signal, causing the whole generation process unstable. To address this challenge, we embed noise along each convolutional layer. In a feature-based generator network, the entire feature map is scaled and biased with the same values. As a result, global effects like shape, illumination or background style could be controlled consistently. Moreover, noise is applied to each pixel individually and thus is eminently suitable for controlling random variations. Once the generative network attempts to control the noise, this leads to spatially inconsistent decisions that will be penalized by the discriminator. Accordingly, TC-GAN can learn to use global and local channels properly without clear guidance.

### Path Length Regularization

Path length regularization makes the network more reliable and makes architectural exploration easier. Specifically, we stimulate fixed-size steps of *W* to generate non-zero fixed-size variations in the image. The bias is measured by observing the corresponding gradient of *W* in the random direction, which should have a similar length regardless of *w* or the image space direction. This indicates that the mapping from potential space to image space is conditional.

At a single *w* ∈ *W*, the local metric scaling properties of the generator mapping *g* (*w*): *W* → *Y* are fixed by the Jacobian matrix *J*_*w*_ = ∂⁡*g*(w)/∂⁡w. Since we wish to preserve the expected length of the vector regardless of its direction, we formulate the regularizer as:


(3) $$E_{w,y∼N\,\left(0,I\right)}\,{\left({∥J_{w}^{T}\,y∥}_{2}-a\right)}^{2}$$

where *y* is a random image with normally distributed pixel intensities, and *w* ∼ *f*(*z*), where *z* are normally distributed. In higher dimensions, this prior is minimized when *J*_*w*_is orthogonal at any *w*. An orthogonal matrix retains length and does not introduce squeezing across any dimension.

This prior is minimized when the expected value of *y* reaches the minimum at each latent space point *w*, respectively, and we start from the internal expectation:


(4) $${ℒ}_{w}:=E_{y}{\left(∥J_{w}^{T}y{∥}_{2}-a\right)}^{2}$$

We use the single-valued decomposition $J_{w}^{T}=U\,\tilde{\Sigma }\,V^{T}$ for analysis. Where *U* ∈ *R^L^*^×*L*^ and *V* ∈ ℝ*^M^*^×*M*^ represent orthogonal matrices. Since rotating a unit normal random variable by an orthogonal matrix will make its distribution invariant, the equation simplifies to:


(5) $${ℒ}_{w}=E_{y}\,{\left({∥U\,\tilde{\Sigma }\,V^{T}\,y∥}_{2}-a\right)}^{2}=E_{y}\,{\left({∥\tilde{E}\,y∥}_{2}-a\right)}^{2}$$

Moreover, the zero matrix effectively marginalizes its distribution in dimension. Then, we simply consider the minimization of the expression:


(6) $${ℒ}_{w}=E_{\tilde{y}}\,{\left({∥\Sigma \,\tilde{y}∥}_{2}-a\right)}^{2}$$

where $\tilde{y}$ is a unit-normal distribution in dimension *L*. All matrices $J_{W}^{T}$ that share the same singular values as Σ generate the same raw loss values. When each diagonal entry of the diagonal matrix Σ is given the specific same value, thus writing the expectation into the integral of the probability density over $\tilde{y}$:


$${ℒ}_{\text{w}}=\int {\left({∥\text{Σ}\,\tilde{\text{y}}∥}_{2}-a\right)}^{2}\,p_{\tilde{\text{y}}}\,\left(\tilde{\text{y}}\right)\,\text{d}\,\tilde{\text{y}}$$

(7) $$={\left(2\,\pi \right)}^{-\frac{L}{2}}\,\int {\left({∥\text{Σ}\,\tilde{\text{y}}∥}_{2}-a\right)}^{2}\,\text{exp}\,\left(-\frac{{\tilde{\text{y}}}^{T}\,\tilde{\text{y}}}{2}\right)\,\text{d}\,\tilde{\text{y}}$$

To observe the radially symmetric form of the density, we alter to polar coordinates $\tilde{y}=r\,\phi$. Such a variable change is replaced by the Jacobian factor *r^L^*^−1^:


(8) $${\tilde{ℒ}}_{w}={\left(2\,\pi \right)}^{-\frac{L}{2}}\,\int_{𝕊}\int_{0}^{\infty }{\left(r\,{∥\Sigma \,\phi ∥}_{2}-a\right)}^{2}\,r^{L-1}\,e\,x\,p\,\left(-\frac{r^{2}}{2}\right)\,d\,r\,d\,\phi$$

where *r* represents the distance from the origin, and ϕ stands for a unit vector. Thus, the ${\left(2\,\pi \right)}^{-L/2}\,r^{L-1}\,\text{exp}\,\left(-\frac{r^{2}}{2}\right)$ denotes the *L*-dimensional unit average density expressed in polar coordinates. The Taylor approximation argument indicates that when *L* is high, the density is well-approximated by density ${\left(2\,\pi \,e/L\right)}^{-\frac{L}{2}}\,\exp\left(-\frac{1}{2}\,{\left(r-\mu \right)}^{2}/\sigma^{2}\right)$for any ϕ. Replacing the density into the integral, the loss is given by approximately:


(9) $${ℒ}_{\text{w}}\approx {\left(2\,\pi \,e/L\right)}^{-L/2}\,\int_{\text{S}}\int_{0}^{\infty }{\left(r\,{∥\text{Σ}\,\phi ∥}_{2}-a\right)}^{2}\,\text{exp}\,\left(-\frac{{\left(r-\sqrt{L}\right)}^{2}}{2\,\sigma^{2}}\right)\,d\,r\,\text{d}\,\phi$$

where the approximation turns out to be exact in the limit of infinite dimension *L*.

By minimizing this loss, we set Σ to obtain a minimum of the function (*r*∥Σϕ∥_2_−*a*)2 over a spherical shell of radius $\sqrt{L}$. According to this function becoming constant in ϕ, the equation reducing to:


(10) $${ℒ}_{w}\approx {\left(2\,\pi \,e/L\right)}^{-L/2}\,𝒜\,\left(S\right)\,a^{2}\,L^{-1}\,\int_{0}^{\infty }{\left(r-\sqrt{L}\right)}^{2}\,e\,x\,p\,\left(-\frac{{\left(r-\sqrt{L}\right)}^{2}}{2\,\sigma^{2}}\right)\,d\,r$$

where ***𝒜***(*S*) indicates the surface area of the unit sphere.

To summarize, we proved that, supposing a high dimensionality *L* of the latent space, the path length prior at each latent space point *w* is minimal if all the singular values of the Jacobian matrix for the generator are equal to a global constant, that is, they are orthogonal up to a global constant. We avoid the explicit computation of the Jacobian matrix by using the same $J_{w}^{T}\,y=\nabla w\,\left(g\,\left(w\right)\cdot y\right)$, and this could be efficiently computed by standard back-propagation. The constant *a* is dynamically set to a long-term exponential moving average of length ${∥J_{w}^{T}\,y∥}_{2}$, enabling the optimization to discover the appropriate global scale on its own.

### Res2Net

**FIGURE 5:**
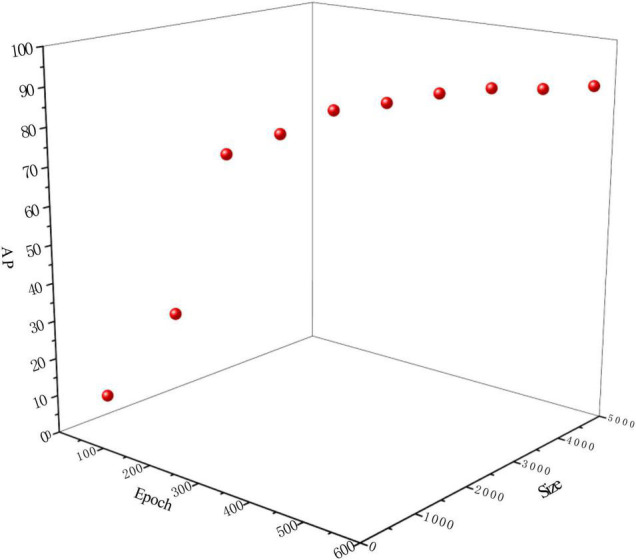
Impact of dataset size and epoch time on TC-GAN.

To alleviate pattern collapse and gradient vanishing, we use a gradient diversion approach (Res2Net) with stronger multi-scale feature extraction capabilities. In essence, a set of 3 × 3 filters are substituted with smaller filter groups, connected in a similar way to the residual mechanism. Figure 5 illustrates Res2Net, we split the feature map uniformly into *s* subsets of feature maps after 1 × 1 convolution, denoted by*xi*, where *i* ∈{1, 2,…*s*}. Each subset of features *x*_*i*_ has the same spatial size compared to the input feature map, but with 1/s number of channels. Besides*x*1, each *x*_*i *_has a corresponding 3 × 3 convolution, denoted by*Ki* (). We denote the output of *K*_*i*_ () by *y*_*i*_. This feature subset is summed with the output of *K*_*i–1*_ () and fed into *K*_*i*_ (). To minimize the parameters and increase *s* simultaneously, we skip the 3 × 3 convolution of *x*_*1*_. Hence, *y*_*i*_could be written as:


(11) $$y_{i}=\left\{ \begin{array}{c} x_{i}\;i=1;\\ K_{i}\,\left(x_{i}\right)\;i=2;\\ K_{i}\,\left(x_{i}+y_{i-1}\right)\,2<i\leq s \end{array}\right.$$

Each 3 × 3 convolutional operator *K*_*i*_ () has the potential to capture feature information from feature splits{*x*_*j*_,*j*≤*i*}. When the feature slice *x*_*j *_ is passed through the 3 × 3 convolution operator, the output may have a larger receptive field than *x*_*j *_. Due to the combinatorial explosion effect, the output of the Res2Net module contains varying amounts and various combinations of receptive field sizes.

In Res2Net, the global and local information of the chrysanthemum image is extracted through processing the splits in a multi-scale approach. To better fuse feature information at different scales, we tandem all the splits and compute them by 1 × 1 convolution. The segmentation and tandem approach allow for efficient convolution operations and feature processing. To minimize the parameter capacity, we skip the convolution of the first segmentation. In this article, we employ *s* to control parameters for the scale dimension. Larger *s* has the potential to allow learning features with richer perceptual field dimensions, with negligible computation of tandem.

### Evaluation Metrics

Average precision (AP) is a common evaluation metric in object detection tasks. In this article, we calculate the average precision (IoU = 0.5) of the tea chrysanthemum to test the performance of the model. The equation is as follows:


(12) $$A\,P=\sum_{k=1}^{N}P\,\left(k\right)\,\Delta \,r\,e\,c\,a\,l\,l\,\left(k\right)$$

where *N* represents the size of the test dataset, *P*(*k*) stands for the precision value of the *k* tea chrysanthemum images, and recall (*k*) denotes the change in recall between *k* and *k*-1 tea chrysanthemum images.

In addition, error and miss rates were introduced in section “Impact of Different Unstructured Environments on the TC-YOLO” to investigate the ability of TC-GAN to generate unstructured environments. error rate indicates a ratio of the number of falsely detected samples to the total samples. miss rate refers to the ratio of undetected samples to the total samples.

### Experimental Setup

The experiments were conducted on a server with an NVIDIA Tesla P100, CUDA 11.2. We built the proposed model using python with the pytorch framework. During training, the key hyperparameters were set as follows: epoch = 500; learning rate = 0.001; and the optimizer used was Adam.

## Results

### Performance of Tea Chrysanthemum – Generative Adversarial Network in Datasets of Different Sizes

To verify the effect of the generated dataset size and the number of training epochs on the chrysanthemum detection task, we randomly selected the datasets with 10 different number of training samples (100, 500, 1000, 1500, 2000, 2500, 3000, 3500, 4000, and 4500) and corresponding ten different training epochs at 100, 200, 250, 300, 350, 400, 450, 500, 550, and 600, respectively, from the generated chrysanthemum dataset and tested them on the proposed TC-YOLO, the results are shown in Figure 5.

It can be seen that the performance of TC-YOLO improves with the increase of the dataset size and training epochs. When the dataset size is less than 1500 and the training epoch is less than 300, the AP value increases rapidly as the dataset size and the training epochs increase (13.54–80.53%, improved by 494.76%). When the dataset size reached 2500, and the training epoch reached 400, the AP values only slightly improved and finally converged (from 87.29 to 90.09%) with the increase of the number of samples and the training epochs. After the dataset size reached 4000 and the training epoch reached 550, the detection performance AP value decreased slightly to 89.51%. Combining these results, we set the optimal dataset size to 3500 and the optimal training epoch to 500 for the test experiments in Sections *B*, *C*, and *D*, as it achieved the highest AP values with the smallest dataset size and the least training epoch.

### Study on the Performance of Traditional Data Enhancement Methods and Tea Chrysanthemum – Generative Adversarial Network

To investigate the performance of classical data enhancement methods and TC-GAN, we selected nine classical data enhancement methods and TC-GAN (Table 3). These data enhancement methods were configured and tested in the TC-YOLO object detection model. The results are shown in Table 3. TC-GAN shows the best performance with an AP value of 90.09%. It was surprising that the advanced data enhancement methods, such as Mixup, Cutout and Mosaic, had a disappointing performance with AP values of only 80.33, 81.86, and 84.31%, respectively. This may be due to the fact that a large amount of redundant gradient flow would greatly reduce the learning capacity of the network. We also found that the performance of Flip and Rotation was second only to TC-GAN, with AP values of 86.33 and 86.96%. The performance of the model improves slightly, with an AP value of 87.39% when Flip and Rotation are both configured on TC-YOLO. Even so, its AP is still 2.7% lower than TC-GAN.

**TABLE 3:** Performance comparison of different data enhancement methods.

Flip	Shear	Crop	Rotation	Grayscale	Blur	Mixup	Cutout	Mosaic	TC-GAN	AP
√										86.33
	√									84.21
		√								83.99
			√							86.96
				√						82.09
					√					80.13
						√				80.33
							√			81.86
								√		84.31
√			√							87.39
									√	90.09

*√ means that this data enhancement method has been adopted.*

### Comparisons With State-of-the-Art Detection Models

To verify the superiority of the proposed model, tea chrysanthemum dataset generated by TC-GAN was used to compare TC-YOLO with nine state-of-the-art object detection frameworks (Kim et al., 2018; Zhang et al., 2018; Cao et al., 2020; Zhang and Li, 2020), and the results are shown in Table 4.

**TABLE 4:** Comparisons with state-of-the-art detection methods.

Method	Backbone	Size	FPS	mAP
RetinaNet	ResNet101	800 × 800	4.54	82.62
RetinaNet	ResNet50	800 × 800	5.31	80.59
RetinaNet	ResNet101	500 × 500	7.23	79.13
RetinaNet	ResNet50	500 × 500	7.87	83.68
EfficientDetD6	EfficientB6	1280 × 1280	5.29	81.23
EfficientDetD5	EfficientB5	1280 × 1280	6.21	83.51
EfficientDetD4	EfficientB4	1024 × 1024	7.93	83.19
EfficientDetD3	EfficientB3	896 × 896	9.28	84.83
EfficientDetD2	EfficientB2	768 × 768	11.66	84.22
EfficientDetD1	EfficientB1	640 × 640	15.26	82.93
EfficientDetD0	EfficientB0	512 × 512	37.61	82.81
M2Det	VGG16	800 × 800	7.08	80.63
M2Det	ResNet101	320 × 320	16.89	85.16
M2Det	VGG16	512 × 512	21.22	80.88
M2Det	VGG16	300 × 300	42.53	78.24
YOLOv3	DarkNet53	608 × 608	12.14	86.52
YOLOv3 (SPP)	DarkNet53	608 × 608	15.66	83.89
YOLOv3	DarkNet53	416 × 416	43.25	84.13
PFPNet (R)	VGG16	512 × 512	24.35	82.41
RFBNetE	VGG16	512 × 512	21.54	77.37
RFBNet	VGG16	512 × 512	45.46	85.53
RefineDet	VGG16	512 × 512	31.33	81.12
RefineDet	VGG16	448 × 448	43.31	79.66
YOLOv4	CSPDarknet53	608 × 608	19.22	85.11
YOLOv4	CSPDarknet53	512 × 512	24.63	84.34
YOLOv5l	CSPDenseNet	416 × 416	42.24	88.83
YOLOv5m	CSPDenseNet	416 × 416	36.91	86.68
YOLOv5x	CSPDenseNet	416 × 416	32.28	84.02
YOLOv5s	CSPDenseNet	416 × 416	47.88	88.29
TC-YOLO	CSPDenseNet	416 × 416	47.53	90.09

**FIGURE 6:**
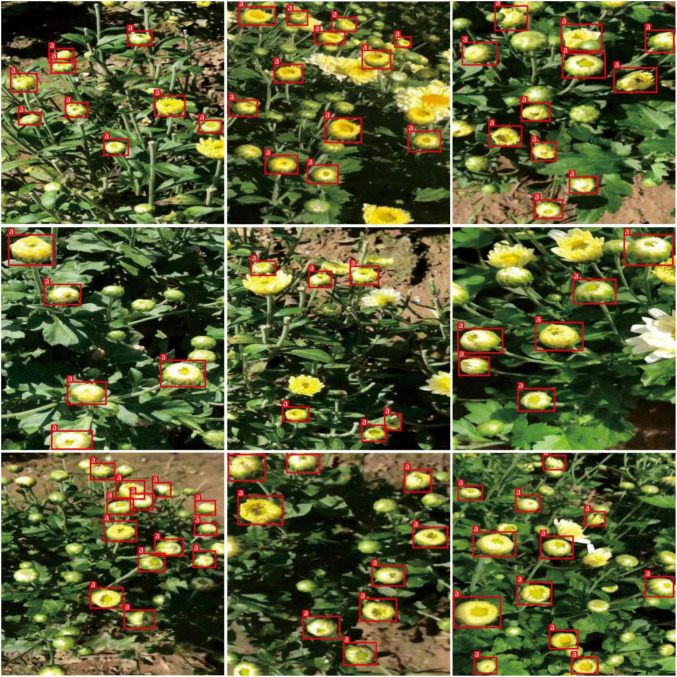
Qualitative results of our method. The red box indicates the recognised tea chrysanthemum.

Table 4 shows that TC-GAN not only achieves excellent performance on the TC-YOLO object detection model with a mAP of 90.09%, but also performs well on other state-of-the-art object detection frameworks. TC-GAN is a general data enhancement method and not constrained to the specific object detectors. Generally speaking, large image sizes benefit model training by providing more local feature information, however, large image sizes (>512 × 512) do not always result in improved performance. In Table 4, all the models with large image sizes (>512 × 512) were unable to achieve a performance above 87%. The main reason for this may be that the image size generated in this article is 512 × 512, which would affect the performance of models requiring a large input size. To match the input size, the images could only be artificially resized to the smaller images, resulting in a reduction in image resolution, and this would considerably affect the final test performance of the models. Also, transfer learning ability varies between models, and this may account for some models with over 512 × 512 resolutions performing poorly. Given the above two reasons, TC-YOLO has relatively better transfer learning ability compared to other object detection models. Therefore, TC-YOLO is used as the test model for generating chrysanthemum images in this article. Besides, TC-YOLO requires the image input size of 416 × 416, making the image resolution a relatively minor impact on the final performance. Furthermore, we deployed the trained TC-YOLO in the NVIDIA Jetson TX2 embedded platform to evaluate its performance for robotics and solar insecticidal lamps systems development. Figure 6 shows the detection results.

### Impact of Different Unstructured Environments on the TC-YOLO

**FIGURE 7:**
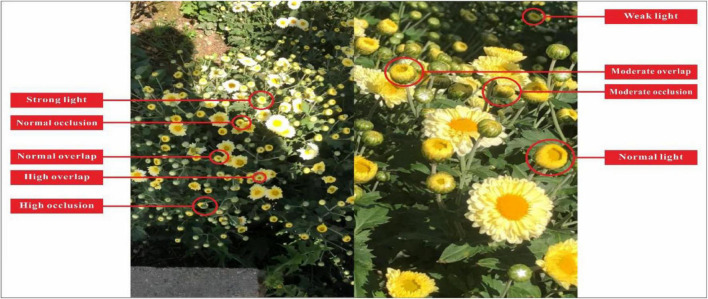
Example of nine unstructured scenarios.

Datasets with complex unstructured environments can effectively improve the robustness of detection models. This study investigated the ability of the proposed TC-GAN to generate complex unstructured environments, including strong light, weak light, normal light, high overlap, moderate overlap, normal overlap, high occlusion, moderate occlusion and normal occlusion, as shown in Figure 7. A total of 26,432 chrysanthemums were at the early flowering stage in the nine unstructured environments. Since there are no mature standards to define these different environments, we set the criteria based on empirical inspection. Strong light is defined as when sunlight obscures more than fifty percent of the petal area. Weak light is defined as when the shadows cover less than fifty percent of the pedal area. Normal light is defined as when the sunlight covers between zero and fifty percent of the petal area. High overlap is defined as when the overlapping area between petals is greater than sixty percent. Moderate overlap is defined when the overlapping area between petals is between thirty to sixty percent. Normal overlap is defined when the overlapping area between petals is between zero to thirty percent. High occlusion is defined as more than sixty percent of the petal area is obscured. Moderate is defined as when thirty to sixty percent of the petal area is obscured. Normal occlusion is defined as when zero to thirty percent of the petal area is obscured. The chrysanthemums are counted separately in different environments. For example, when chrysanthemums in normal light, normal overlap and normal occlusion appear in one image simultaneously, their numbers increase by one in the calculation.

Table 5 shows that under normal conditions, with normal light, normal overlap and normal shading, the AP values reached at 93.43, 94.59, and 94.03%, respectively. When the unstructured environment became complicated, the AP values dropped significantly, especially under the strong light environment, with only 77.12%. AP value. Intriguingly, the error rate (10.54%) was highest under the strong light, probably because the light added shadows to the chrysanthemums. It also may be due to the poor ability of TC-GAN to generate high quality images under light environment. The high overlap had the highest miss rate of 13.39%. Furthermore, overall, overlap had the least influence on the detection of chrysanthemums at the early flowering stage. Under high overlap, the AP, error and miss rates were 79.39, 7.22, and 13.39%, respectively. Illumination had the biggest effect on chrysanthemum detection at the early flowering stage. Under high light, the accuracy, error and miss rates were 77.12, 10.54, and 7.25%, respectively.

**TABLE 5:** Impact of different unstructured scenarios on the TC-YOLO.

Environment	Count	Correctly identified		Falsely identified		Missed	
				
		Amount	Rate (%)	Amount	Rate (%)	Amount	Rate (%)
Strong light	6511	5021	77.12	686	10.54	804	7.25
Weak light	10162	8786	86.46	857	8.43	519	5.11
Normal light	18686	17458	93.43	988	5.29	240	1.28
High overlap	5249	4167	79.39	379	7.22	703	13.39
Moderate overlap	11892	10420	87.62	659	5.54	813	6.84
Normal overlap	17443	16499	94.59	419	2.4	525	3.01
High occlusion	7811	6284	80.45	729	9.33	798	10.22
Moderate occlusion	12162	10661	87.66	630	5.18	890	7.16
Normal occlusion	19299	18147	94.03	648	3.36	504	2.61

### Comparison of the Latest Generative Adversarial Neural Networks

To fully investigate the performance of TC-GAN, TC-GAN and 12 state-of-the-art generative adversarial neural networks were tested on the chrysanthemum dataset using the TC-YOLO model. The proposed TC-GAN generated chrysanthemum images with a resolution of 512 × 512. However, there is variability in the resolution of the generated images from different generative adversarial neural networks. Therefore, to facilitate testing of the TC-YOLO model and to ensure a fair competition between TC-GAN and these generative adversarial neural networks, we modified the output resolution of the latest generative adversarial neural networks. According to the original output resolution of these neural networks, we modified the output resolution of LSGAN, Improved WGAN-GP to 448 × 448, BigGAN kept the original resolution unchanged, and the output resolution of the remaining generative adversarial neural networks were all adjusted to 512 × 512, while other parameters were kept fixed. The performance is shown in Table 6.

**TABLE 6:** Comparison between tea chrysanthemum – generative adversarial network (TC-GAN) and state-of-the-art GANs.

Method	Size	Times/min	AP
Improved SN-GAN	32 × 32	1290	80.61
BigGAN	512 × 512	1610	86.45
Dist-GAN	64 × 64	1322	80.68
Progressive GAN	64 × 64	1256	81.11
LSGAN	112 × 112	1410	84.03
Rob-GAN	128 × 128	1293	85.28
MGAN	64 × 64	1151	82.39
AutoGAN	64 × 64	1340	83.25
Improved DCGAN	64 × 64	1280	84.38
DAG	48 × 48	1768	83.29
Improved WGAN-GP	28 × 28	1640	76.16
Improved WGAN	128 × 128	1501	87.16
TC-GAN	512 × 512	1460	90.09

Table 6 shows some experimental details. TC-GAN has the best performance among the latest 12 generative adversarial neural networks, with an AP value of 90.09%. It is worth noting that TC-GAN does not have an advantage in training time among all the latest generative adversarial neural networks, with all nine models training faster than TC-GAN. Only BigGAN, Improved WGAN-GP and Improved WGAN are slower than TC-GAN, with training times of 50, 180, and 241 min slower than TC-GAN, respectively. This may be due to the design of the network structure, which increases the depth of the network and adds a gradient penalty mechanism. In contrast to most convolutional neural networks, deepening the structure of generative adversarial neural networks tends to make training unstable. Also, the gradient penalty mechanism is very sensitive to the choice of parameters, and this helps training initially, but subsequently becomes difficult to optimize. Furthermore, in general, the smaller the original generated image size, the worse the performance of the generative adversarial neural network in the detection task. This is because, firstly, current mainstream adversarial neural networks generate images with low resolution, and artificially enlarging the resolution would blur the image, thus affecting the detection accuracy. Then, some latest models, such as Progressive GAN, Improved DCGAN and so on, are designed for better faces, and these models are not robust in terms of transfer ability. Interestingly, among the 12 latest generative adversarial neural networks, most of the network structures are unconditional. Nevertheless, from a comprehensive performance perspective, network structures with conditional mechanisms, such as the improved WGAN, have surprisingly good performance. Its training time is only 41 min slower than TC-GAN, while the AP value is only slightly lower by 2.93%. Network structures with conditional mechanisms are undoubtedly valuable to learn from, and adding conditional mechanisms could be a future direction to improve the performance of TC-GAN. To visualize the performance of TC-GAN, the images generated by TC-GAN are shown in Table 7.

**TABLE 7:** Generation results of different GANs.

Methods	Result
Improved WGAN-GP	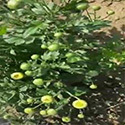 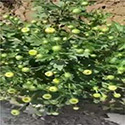 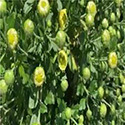 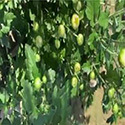
SN-GAN	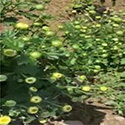 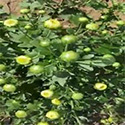 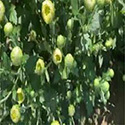 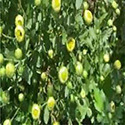
Dist-GAN	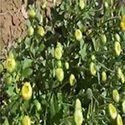 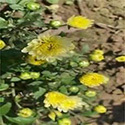 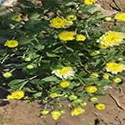 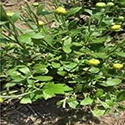
Progressive GAN	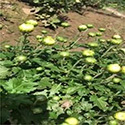 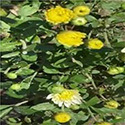 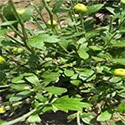 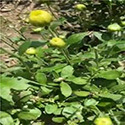
MGAN	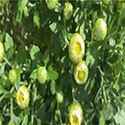 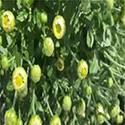 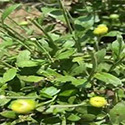 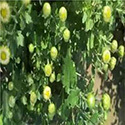
AutoGAN	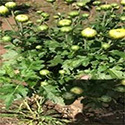 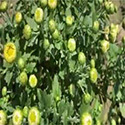 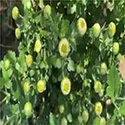 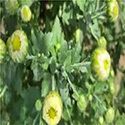
DAG	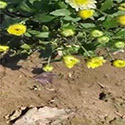 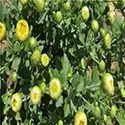 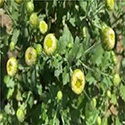 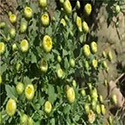
LSGAN	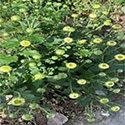 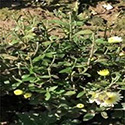 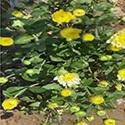 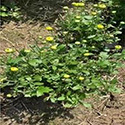
Improved DCGAN	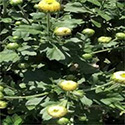 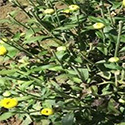 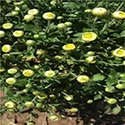 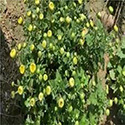
Rob-GAN	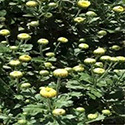 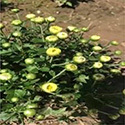 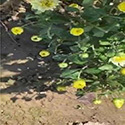 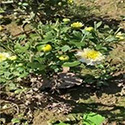
BigGAN	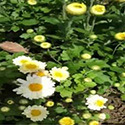 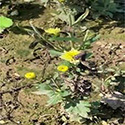 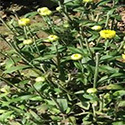 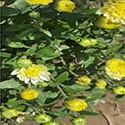
Improved WGAN	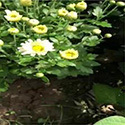 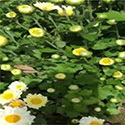 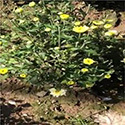 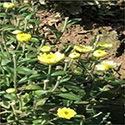
TC-GAN	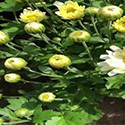 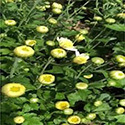 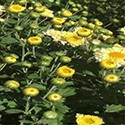 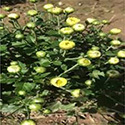

## Discussion

**FIGURE 8:**
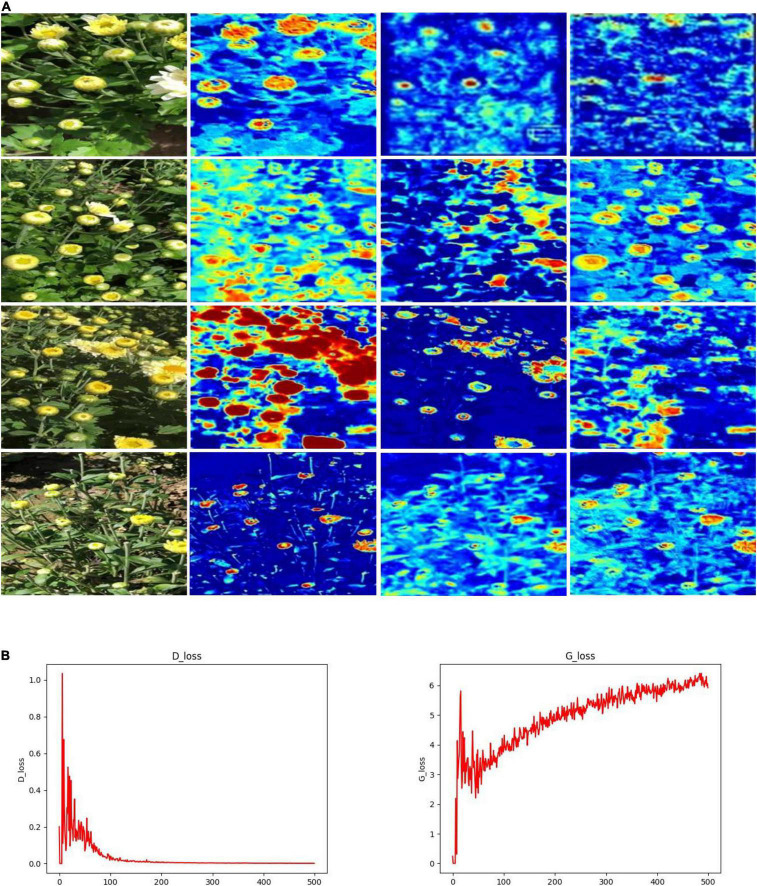
**(A)** Visualization results and **(B,C)** training process.

To investigate the three issues summarized in the section “Introduction,” we proposed the TC-YOLO and compared its results with the related work in Table 2. Our proposed TC-GAN generates high resolution images (512 × 512), and the *E* section of the experimental results shows that high resolution images can significantly enrich environmental features and thus improve the robustness of the model. GAN is prone to pattern collapse and gradient vanishing during the training process, resulting in the lack of diversity in the generated image features (Wang et al., 2021). TC-GAN is able to generate images containing complex unstructured environments including illumination, overlap and occlusion to gain the benefit for detection under field environments, whereas most of synthetic images generated from other GANs listed in Table 2 provide limited diversity and clear backgrounds. To intuitively view the image features through the generation process, we show the visualization process and training process in TC-YOLO (Figure 8). It can be seen that the important part (flower heads) of the plants is clearly activated and captured with the TC-YOLO.

There are several points to be optimized for TC-GAN despite its good detection performance. First, currently, there are no suitable metrics to evaluate synthetic images. FID is a widely recognized metric for evaluating synthetic images, but the FID metric is dedicated to evaluating several specific datasets and is not applicable to customized datasets. We can only evaluate the quality of synthetic images by their detection results in an object detection model. Therefore, establishing a standard set of evaluation metrics is an urgent issue to be addressed. Next, the training cost of TC-GAN is expensive. As can be seen from Table 6, the training of the whole model takes 1460 min under the 16 GB video memory of Tesla P100, and an ordinary device is difficult to train effectively. Thus, the light weight of TC-GAN is beneficial to the promotion of the technology. Besides, according to the experimental results in section “Impact of Different Unstructured Environments on the TC-YOLO” of the experimental results, TC-GAN can not fully construct images well for the illumination environmental setting. Note that the lack of efficient interaction between the generator network and the discriminator network leads to constant oscillation in the gradient and difficulty in convergence, as shown in Figure 8B. This is still a challenge without fully addressed in generative adversarial networks, and we suggest more attention should be paid to solve this challenge. Finally, our proposed model was deployed in NVIDIA Jetson TX2 with approximately 0.1 s per chrysanthemum inference time (the image size is 416 × 416). It is not real-time performance, and this deserves further optimization for network architecture such as network pruning and quantization.

## Conclusion

This article presents a novel generative adversarial network architecture TC-GAN for generating tea chrysanthemum images under unstructured environments (illumination, overlap, occlusion). The TC-YOLO model is able to generate images with a resolution of 512 × 512 and achieves the AP of 90.09%, showing supreme results with other state-of-the-art generative adversarial networks. Finally, we deployed and tested the TC-YOLO model in the NVIDIA Jetson TX2 for robotic harvesting and solar insecticidal lamps systems development, achieving approximately 0.1 s per image (512 × 512). The proposed TC-GAN has the potential to be integrated into selective picking robots and solar insecticide lamp systems via the NVIDIA Jetson TX2 in the future.

## Data Availability Statement

The original contributions presented in the study are included in the article, further inquiries can be directed to the corresponding authors.

## Author Contributions

CQ: conceptualization, methodology, software, writing – original draft, and writing – review and editing. JG: conceptualization and writing – review and editing. KC: supervision and writing – review and editing. LS: supervision, project administration, funding acquisition, and writing – review and editing. SP: writing – review and editing. All authors contributed to the article and approved the submitted version.

## Conflict of Interest

The authors declare that the research was conducted in the absence of any commercial or financial relationships that could be construed as a potential conflict of interest.

## Publisher’s Note

All claims expressed in this article are solely those of the authors and do not necessarily represent those of their affiliated organizations, or those of the publisher, the editors and the reviewers. Any product that may be evaluated in this article, or claim that may be made by its manufacturer, is not guaranteed or endorsed by the publisher.
